# Common physiologic and proteomic biomarkers in pulmonary and coronary artery disease

**DOI:** 10.1371/journal.pone.0264376

**Published:** 2022-03-09

**Authors:** Andreas Casselbrant, Artur Fedorowski, Sophia Frantz, Gunnar Engström, Per Wollmer, Viktor Hamrefors

**Affiliations:** 1 Department of Clinical Sciences, Lund University, Malmö, Sweden; 2 Department of Oncology, Skåne University Hospital, Lund, Sweden; 3 Department of Cardiology, Karolinska University Hospital, Stockholm, Sweden; 4 Department of Translational Medicine, Lund University, Malmö, Sweden; 5 Department of Medical Imaging and Physiology, Skåne University Hospital, Malmö, Sweden; 6 Department of Internal Medicine, Skåne University Hospital, Malmö, Sweden; Scuola Superiore Sant’Anna, ITALY

## Abstract

**Objective:**

Chronic obstructive pulmonary disease (COPD) and coronary artery disease (CAD) are leading causes of global morbidity and mortality. There is a well-known comorbidity between COPD and CAD, which is only partly explained by smoking and other known common risk factors. In order to better understand the relationship between COPD and CAD, we analyzed myocardial perfusion, pulmonary function and novel cardiovascular biomarkers in patients with symptoms suggesting myocardial ischemia.

**Methods:**

A total of 396 subjects from the Swedish Biomarkers and Genetics CardioPulmonary Physiology Study (BiG CaPPS) were included, all of whom had been referred to myocardial perfusion imaging due to suspected myocardial ischemia. Subjects performed myocardial perfusion imaging (MPI), pulmonary function tests (PFT) and analysis of 92 proteomic biomarkers, previously associated with cardiovascular disease. Linear regression was used to study the relationship between MPI and PFT results and proteomic biomarkers.

**Results:**

Subjects with CAD (n = 159) had lower diffusing capacity (D_LCO_) than patients without CAD (6.64 versus 7.17 mmol/(min*kPa*l); p = 0.004) in models adjusted for common covariates such as smoking, but also diabetes and brain natriuretic peptide (BNP). The association remained significant after additional adjustment for forced expiratory volume in one second (FEV_1_) (p = 0.009). Subjects with CAD, compared with subjects without CAD, had higher total airway resistance (0.37 vs 0.36 kPa/(l/s); p = 0.036). Among 92 protein biomarkers, nine were associated with a combined diagnosis of CAD and airflow obstruction: *VSIG2*, *KIM1*, *FGF-23*, *REN*, *XCL1*, *GIF*, *ADM*, *TRAIL-R2* and *PRSS8*.

**Significance:**

Diffusing capacity for carbon monoxide is decreased in patients with CAD, independently of decreased FEV_1_, diabetes, and elevated BNP. Several cardiovascular biomarkers are associated with co-existent CAD and airflow obstruction, but none with airflow obstruction only. The current findings indicate that the interaction between CAD and lung function is complex, including mechanisms beyond the known association between CAD and reduced ventilation.

## Introduction

Chronic obstructive pulmonary disease (COPD) and atherosclerotic cardiovascular disease, most notably coronary artery disease (CAD), are leading causes of death world-wide [1]. Studies have consistently shown a strong association between COPD and CAD [2–5]. Reduced lung function, including low forced expiratory volume in one second (FEV_1_) and low forced vital capacity (FVC), as well as a manifest diagnosis of COPD are all associated with CAD [6–8].

Whereas the strong relationships that exist between reduced lung function, COPD and CAD have not yet been fully understood from a pathophysiological view, a number of underlying mechanisms have been suggested. One such mechanism is low-grade systemic inflammation, which is known to play a role in the pathogenesis of both diseases [9–11]. Such inflammation may potentially damage elastin in the arteries as well as in the alveoli, which may in turn cause arterial wall stiffness and loss of functional alveolar tissue [12–15], providing an explanation for the increased arterial stiffness that could be observed in COPD [16,17]. Moreover, such inflammation may cause endothelial dysfunction in both the coronary and the pulmonary vasculature [18], providing another possible link between coronary and pulmonary disease. Smoking increases inflammation and is regarded as the most important common risk factor for both COPD and CAD [19,20], although about 25% of patients with COPD report no history of smoking [21]. Other suggested common risk factors for COPD and CAD include airborne pollution [22–24], cardiovascular autonomic dysfunction [25], and genetic factors [26].

Accounting for the strong relationship that exist between COPD and CAD, further studies of specific lung function parameters and their relationships with CAD may further aid in understanding the links between the diseases.

First, whereas previous studies examined predominantly the relationships between CAD and conventional spirometry measurements such as FEV_1_ and VC, other lung function measurements such as diffusing capacity for carbon monoxide (D_LCO_) and impulse oscillometry (IOS), have been less extensively studied. These measurements do not represent the sole existence of bronchial disease, but may point to other pulmonary pathology that may represent an additional link with CAD [27]. D_LCO_ is estimated by the difference in partial pressure of carbon monoxide in inhaled and air exhaled after a 10 seconds breath-hold. A difference lower than normal implies a reduced carbon monoxide uptake in the lung, which provides a valid estimate of the oxygen diffusion. D_LCO_ is used to assess the function of the alveolar-capillary membrane, and both decreased alveolar surface area and increased thickness of the blood-air barrier will impair the diffusion of gases and lower the D_LCO_. Lung fibrosis, heart failure, anemia and increased pressure in the pulmonary circulation also affect D_LCO_ [28–30]. IOS measures mechanical properties of the lung by the application of a sound wave. It has been shown to detect subtle changes in a patients airway function earlier than with the conventional spirometry, and has been proven to detect small airway disease in symptomatic patients with normal spirometry [31–33]. Thus, pathological values of D_LCO_ and IOS in patients with CAD, may indicate potential mechanisms such as concurrent damage to elastin in the artery and the alveoli [12–15] as well as potential endothelial dysfunction in the coronary and pulmonary vasculature [18].

In addition to specific lung function measurements, the analysis of protein biomarkers with potential involvement in the pathogenesis of both COPD and CAD, may reveal novel common pathophysiological pathways. Whereas a large number of such biomarkers have been separately identified for COPD [34,35] and CAD [36], studies examining potential association between such biomarkers and both COPD and CAD are sparse. Inflammatory cytokines such as *Interleukin-6 (IL-6)*, *Interleukin-8 (IL-8)*, *Tumor necrosis factor (TNF-alpha)*, *Monocyte chemoattractant protein 3 (MCP-3)* and *C-C Motif chemokine ligand 11 (CCL11)* have been shown to be increased in both diseases [37–39], in turn supporting the hypothesis that inflammation may be a treatable target in both diseases [40,41]. In addition to inflammatory pathways, the identification of additional plasma-biomarkers that associate with both diseases, may give clues to other common pathways. As a complement to genetic studies revealing common genetic variants for CAD and COPD [26] the study of such common biomarkers may ultimately reveal novel potential targets for the prevention and treatment of both COPD and CAD.

Thus, in order to expand our knowledge of the relationships between CAD and COPD, we aimed to assess:

1. The relationship between CAD and specific physiological measurements from extended pulmonary function testing (primary objective) and
2. the relationship between cardiovascular proteomic biomarkers and airflow obstruction with or without concurrent CAD (secondary objective) in patients evaluated for suspected angina pectoris.

## Methods

### Study population

#### The BiG CaPPS cohort

Data were collected from the Biomarkers and Genetics CardioPulmonary Physiology Study (BiG CaPPS)-cohort, which has been previously described in detail [42]. In short, the BiG CaPPS cohort consists of 500 outpatients with suspected stable myocardial ischemia, all of whom had been clinically referred by a physician to myocardial perfusion imaging (MPI) during 2014–2017. Except for inability to provide written informed consent, there were no further exclusion criteria in BiG CaPPS. The 500 subjects that constitute the BiG Capps cohort were selected from a total of 847 eligible subjects that were invited to participate at the time of MPI (59% participation). No data was obtained from patients who declined participation. At the time of enrollment, which occurred on the same day as the MPI, the subjects filled in a questionnaire including medical history, medications, and a detailed assessment of smoking history. Information regarding diagnoses hypertension and diabetes was obtained from the questionnaire, i.e. a positive response to the question “Have you been diagnosed and/or treated for any of the following: High blood pressure; Have you been diagnosed and/or treated for any of the following: Diabetes”. Similarly, smoking history was self-reported by participants answering the question " Have you ever smoked regularly for one month or more?" in the questionnaire. Current smoking was defined as smoking daily for one month within the last year. Former smoker was defined as regular smoking for at least one month but not within the last year. Resting blood pressure and heart rate were recorded once prior to MPI, using calibrated standard blood pressure equipment with automatic reading, as part of a local, unpublished clinical protocol for the MPI at the clinic. In addition, blood sampling was done during the preparations for the MPI and the blood was handled by the local Clinical Research Unit at Skåne University Hospital. Relative concentrations of brain natriuretic peptide (BNP), used in analyses as a proxy of heart failure, were obtained from the blood samples. Study participants were not required to fast prior to blood sampling. In addition to the MPI, study participants performed pulmonary function tests (dynamic spirometry and IOS) as part of the study within one month from the date of the MPI. The examinations were conducted at the Department of Clinical Physiology and Nuclear Medicine at Skåne University Hospital in Malmö, Sweden, between June 2014 and October 2017. MPI, pulmonary function test, blood pressure measurements and blood sampling were performed by clinical personnel working at the department. The equipment used was calibrated for clinical use. All participants provided informed written consent. The study was done in accordance with the Declaration of Helsinki and was approved by the Regional Ethics Board in Lund (DNR 2013/242).

#### The subset of the BiG CaPPS cohort included in the current study

**Fig 1:**
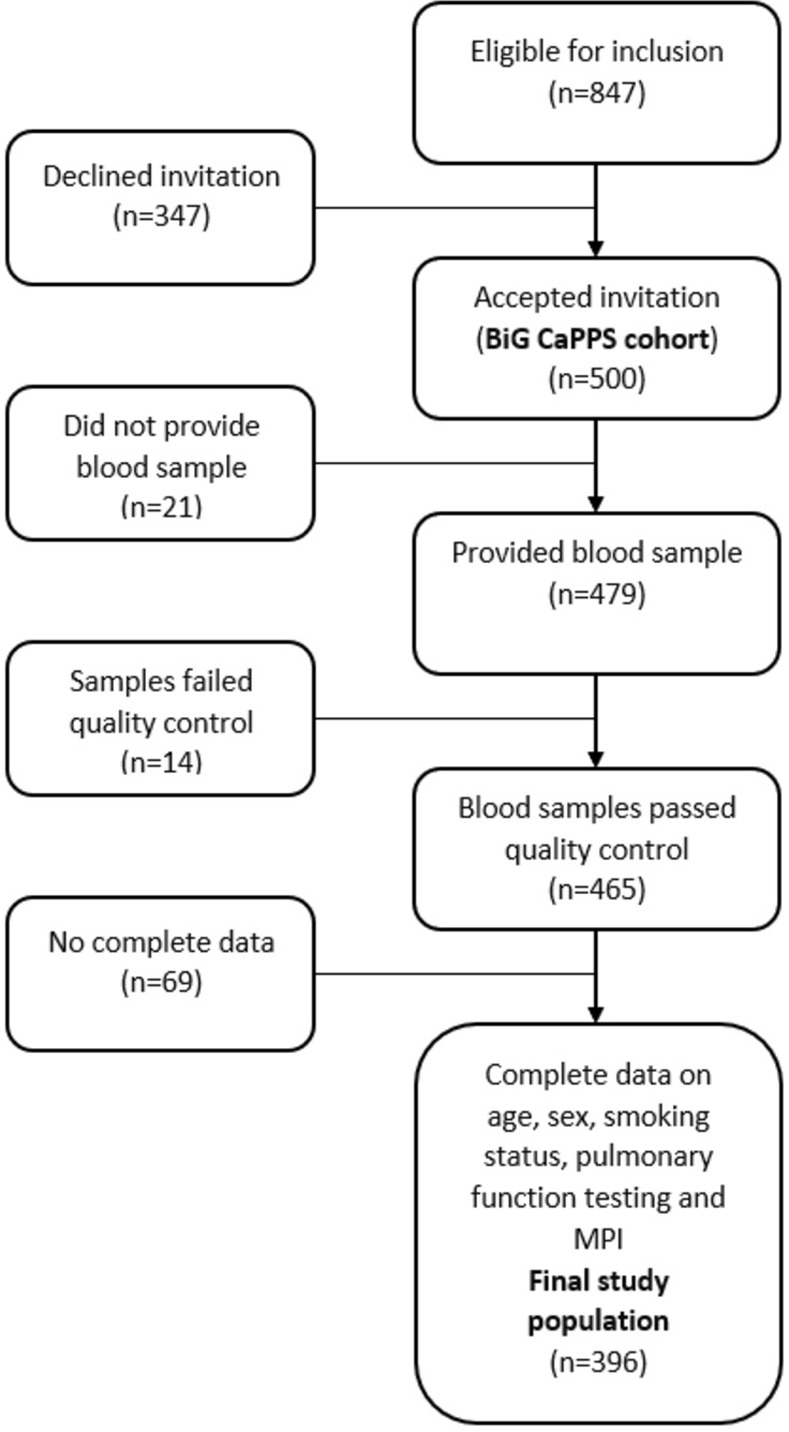
Legend: Study flowchart. The selection of patients for the current study. BiG CaPPS = Biomarkers and Genetics CardioPulmonary Physiology Study.

Out of 500 participants that were enrolled into the BiG CaPPS cohort, 479 provided blood samples. A total of 465 of these blood samples could be successfully analyzed by Olink (for criteria, see below). For the current study, complete data on age, sex, smoking status, pulmonary function testing and MPI were criteria for inclusion in the analyses, leaving 396 subjects that were included in the final study population (Fig 1).

### Myocardial perfusion imaging

Myocardial perfusion imaging (MPI) was performed and interpreted according to current guidelines [43,44] as a part of routine clinical investigation at the Department of Clinical Physiology and Nuclear Medicine at Skåne University Hospital in Malmö, Sweden. The MPI protocol, which has been previously described in detail [42], included provocation with either exercise on a bicycle or pharmacological provocation with adenosine or regadenosone, after which ^99m^Tc-tetrofosmin was injected, and Single Photon Emission Computed Tomography (SPECT) Images were obtained using a dual-head gamma camera (Siemens AG Medical Solutions, Erlangen, Germany). From March 2015 the SPECT acquisition was complemented by a low dose CT scan for attenuation correction.

### Pulmonary function testing

Pulmonary function testing (PFT), including dynamic spirometry after bronchodilation, measurement of carbon monoxide diffusing capacity (D_LCO_), as well as IOS measurements (described in detail below), were performed on a separate day within one month after the MPI examination. Subjects were asked to refrain from smoking for four hours before the examination. Objective data on whether participants actually did refrain from smoking were not available. The examination was performed according to clinical guidelines [45] at the Department of Clinical Physiology at Skåne University Hospital SUS Malmö using standard equipment (Masterscreen PFT and IOS, Jaeger, Würzburg, Germany.) Spirometry and measurements of D_LCO_ (including calibrations) were performed according to the standards issued by the American Thoracic Society and the European Respiratory Society [45,46] and IOS measurements according to the recommendations by Oostveen et al [47]. All examinations were performed by locally certified technicians/biomedical scientist with special expertise in PFT. Of note, the same technicians/biomedical scientist also performed all the clinical PFT examinations (more than 1000 yearly clinical examinations) at the time of the study.

IOS measures mechanical properties of the lung by the application of a sound wave, superimposed on the subject’s tidal breathing, that travels down the respiratory tract. The ratio between measured pressure and flow due to the sound wave, separated from the tidal breathing, describes the respiratory impedance. Impedance can be interpreted as the sum of resistance and reactance opposing the applied pressure impulses from the sound wave and is measured with several frequencies between 5 and 35 Hz. Low frequency oscillations travel deep into the lung and are believed to give information from the whole respiratory system while higher frequencies are assumed to characterize the larger airways. The in-phase component of the impedance signal is interpreted as the respiratory resistance (R) which gives information about the forward pressure of the conducting airways, while the out of phase component is termed the respiratory reactance (X), which is a sum of mass-inertive and capacitive forces. Resistance at low frequency (R5) is often assumed to represent the entire bronchial tree, whereas resistance at higher frequency (R20) is assumed to reflect resistance n proximal airways. Therefore, R5-R20 is often taken to represent peripheral airways [31]. As indicated in the introduction, IOS has been shown to detect subtle changes in a patients airway function earlier than with the conventional spirometry, and has been proven to detect small airway disease in symptomatic patients with normal spirometry [31–33].

### Cardiovascular biomarkers

Blood sample analysis was performed by Olink Proteomics, using Proximity Extension Assay technology. Plasma biomarkers were measured from supine blood samples (total volume: 30 ml) that were centrifuged, then stored as 16 × 250 μL aliquots of EDTA plasma in plastic thermotubes, and frozen at -80°C. The samples were then thawed before biomarker analysis. Samples were analyzed by Olink Proteomics using the Cardiovascular II panel, consisting of 92 cardiovascular protein biomarkers. Proteins for this panel were selected by Olink in consultation with key opinion leaders in cardiovascular proteomics (https://www.olink.com/products/target/cvd-ii-panel/). Quality control was assured by Olink Proteomics, who uses a technique of Normalised Protein Expression (NPX). In short, four internal controls are added to each sample to monitor the quality of assay performance, as well as the quality of individual samples. The quality control is performed in two steps: In the first step, each sample plate is evaluated on the standard deviation of the internal controls. This should be below 0.2 NPX. Only data from sample plate that pass this quality control will be reported. In the second step the quality of each sample is assessed by evaluating the deviation from the median value of the controls for each individual sample. Samples that deviate less than 0.3 NPX from the median pass the quality control. In total, 479 patients from the BiG CaPPs cohort were analyzed, of which 465 samples (97%) passed quality control. Only samples that passed quality control were included in the current study. The Intra- and Inter-Assay CV were 6 and 11% respectively.

### Airflow obstruction and CAD definitions

Airflow obstruction was defined according to spirometry: Ratio of forced expiratory volume in one second (FEV_1_) to vital capacity (VC) < 0.70, after administration of a bronchodilator. Moreover, all tests, including visual flow-volume curves were interpreted by one of two physicians trained in lung function testing (VH, PW).

Established CAD (hereafter termed “CAD”) was defined as previous myocardial infarction, angina pectoris, confirmed significant coronary atherosclerosis on previous imaging, or confirmed myocardial ischemia on MPI performed as part of the current study. Information on participants previous diagnoses and imaging were collected from the referrals for MPI and/or from the questionnaires that each study participant filled out at the time of inclusion. In case of uncertainty, medical records were studied in order to retrieve the data. Patients claiming to have angina on the questionnaire but without a confirmed diagnosis of angina and with no evidence of ischemia on MPI (n = 13), were classified as non-CAD.

### Statistics

#### Lung function measurements in relation to CAD status

Separate linear regression models were created for each of the individual physiological lung measurements of interest (dependent variable). The lung function measurements were *VC*, *FEV*_*1*_, *D*_*LCO*_ *and IOS (Resistance [R] 5Hz*, *R20Hz*, *R5-20Hz*, *Area of reactance [AX])*. The absolute values of IOS measurements (except 5Hz-20Hz difference) were log-transformed to achieve normal distribution. CAD was the independent variable in all models, in addition to covariates age, sex, height, smoking, diabetes and hypertension (Minimally adjusted model). BNP was added to the minimally adjusted model to create Adjusted model 1. There are known relationships between CAD and FEV_1_ [8], CAD and COPD [6], as well as COPD and D_LCO_ [48]. In order to assess the association between CAD and D_LCO_ independently of these relationships, an additional model for D_LCO_ (Adjusted model 2) was created where adjustment for FEV_1_ was added to Adjusted model 1. BNP measurements were received as relative concentration from Olink and were not normally distributed. These data were therefore grouped into quartiles before analyses were performed. BNP was included in the model not as a confounder but because the authors wanted to estimate the effect of CAD on lung function parameters that is independent of heart failure, which is clinically well known to be associated with reduced lung function [49,50]. A directed acyclic graph is provided in the Supplementary data (Supplementary Figure 1 in S1 Data). Finally, as a supplementary analysis, CAD was substituted for the functional variable “coronary ischemia on MPI” in the adjusted model. The model assumptions were reviewed visually by histogram to ensure normal distribution. A power calculation using PS Power (Developed by William D. Dupont and Walton D. Plummer, Vanderbuilt University; https://biostat.app.vumc.org/wiki/Main/PowerSampleSize) was done based on detecting a difference (i.e. beta) in D_LCO_ of 0.5 mmol, with an standard error of the regression errors of 1,3 and a standard deviation of the independent variable of 0,49. In order to achieve a power of 0,8, we would need 223 subjects, which was well exceeded in the current study.

#### Proteomic biomarkers in relation to CAD status and airflow obstruction

Proteomic biomarkers were compared in patients with diagnoses against patients without diagnoses, according to groups *airflow obstruction* vs *without airflow obstruction*, *CAD* vs *without CAD*, *combined diagnosis (airflow obstruction and CAD)* vs *without combined diagnosis*, *and ischemia on MPI* vs *without ischemia on MPI*. Linear models were selected after consultation by statistical expertise within Olink and the model assumptions were checked by visual inspection. The linear regression analyses were adjusted for age, sex and smoking status, with biomarker value as the dependent value. P-values were adjusted for multiple testing using the Benjamini-Hochberg method. Plasma biomarkers are not presented as absolute concentrations, but as relative concentrations in the Normalised Protein Expression (NPX) unit, generated on a log2 scale.

Baseline characteristics (Table 1) are presented as mean (SD) unless otherwise specified.

**Table 1:** Patient characteristics.

	All patients (n = 396)	No diagnosis (n = 149)	AO only (n = 88)	CAD only (n = 103)	Combined AO and CAD (n = 56)
Age, years	66.4 (10.2)	63.4 (11.6)	68.4 (8.9)	66.3 (9.3)	71.5 (7.2)
Sex, % women	46.7	55.7	59.1	27.2	39.3
Smoking status, %					
Never smoker	33.1	46.3	28.4	26.2	17.9
Ex-smoker	53.0	45.0	50.0	62.1	62.5
Current smoker	13.9	8.7	21.6	11.7	19.6
Diabetes, %	22.0	15.4	12.5	33.0	33.9
Hypertension, %	57.6	50.3	61.4	67.0	53.6
Heart rate, min^-1^	69.5 (11.7)	69.0 (11.5)	72.7 (13.2)	68.3 (11.0)	68.0 (10.3)
Systolic blood pressure, mmHg	138.9 (17.4)	138.0 (18.1)	140.8 (18.3)	138.9 (16.7)	138.5 (16.0)
Diastolic blood pressure, mmHg	79.7 (8.9)	80.0 (9.6)	81.2 (7.9)	79.3 (8.9)	77.5 (8.4)
Known CAD at time of inclusion, %	34.1	-	-	79.6	94.6
Known COPD at time of inclusion, %	8.1	1.3	15.9	3.9	21.4

Displayed as mean (SD) unless otherwise specified.

CAD = coronary artery disease; COPD = Chronic obstructive pulmonary disease; AO = airflow obstruction.

Current smoking = Smoking daily for one month during the last year.

Ex-smoker = Earlier daily smoking for at least one month and no smoking during the last year.

Never smoker = Never smoking daily for one month.

Statistics were done by SPSS Statistics 25.0 from IBM Corp. (SPSS Inc., Chicago, IL, USA).

## Results

Three-hundred-and-ninety-six patients that were included in the final study population were middle aged on average and slightly more men than women were included. Approximately, one third of the subjects had previously known CAD, whereas eight percent had known COPD prior to inclusion in the study. Of note, following inclusion in the study only 14% had reversible ischemia on MPI, whereas 36% had airflow obstruction according to spirometry criteria. Characteristics of the 396 patients that were included in the final study population are listed in Table 1.

### Pulmonary function test parameters in relation to CAD

**Fig 2:**
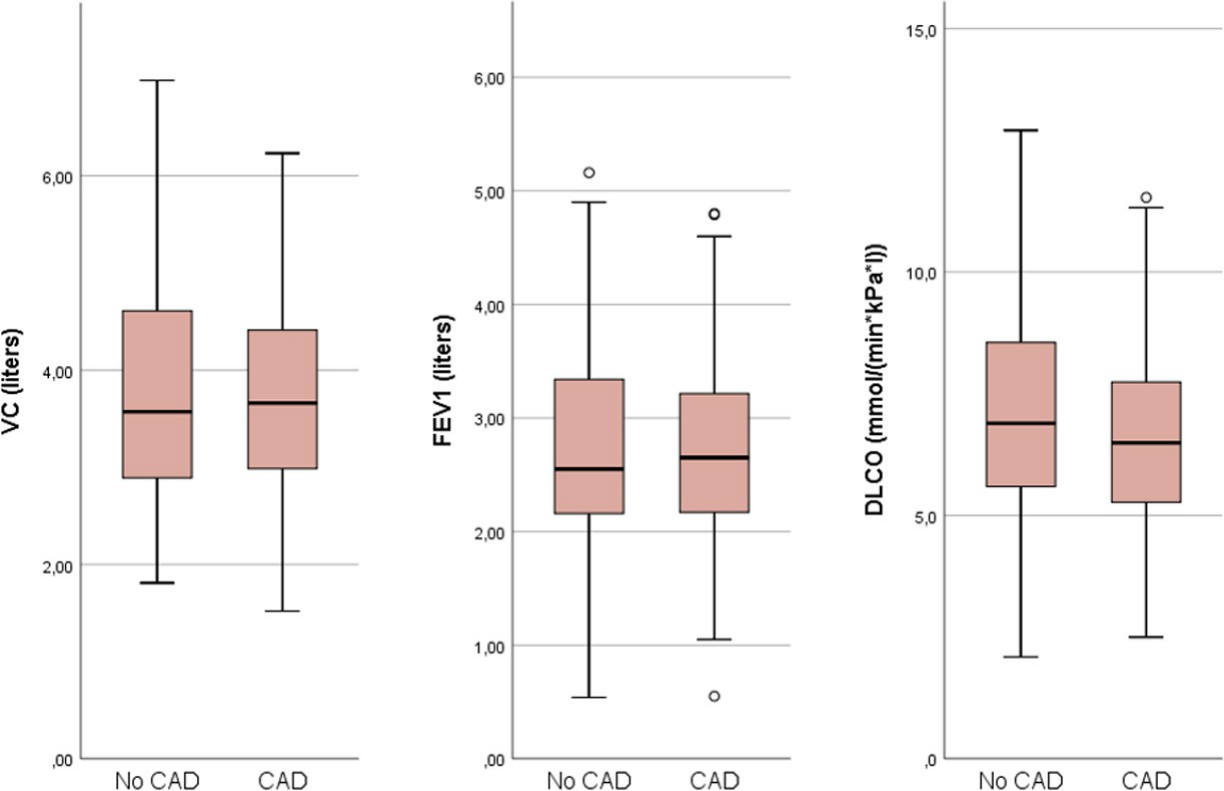
Lung function measurements in non-CAD vs CAD. VC = vital capacity; FEV_1_ = forced expiratory volume in one second; D_LCO_ = diffusing capacity for carbon monoxide.

Patients with CAD had lower VC and D_LCO_ compared with patients without CAD (Fig 2 and Table 2). The association between D_LCO_ and CAD was significant also after adjustment for FEV_1_ (p = 0.009). CAD was also significantly associated with an increased airflow resistance at 5 Hz. There was no association between CAD and resistance at 20Hz or frequency-dependent resistance (5Hz-20Hz). CAD was associated with an increase in reactance area (AX).

**Table 2:** Mean spirometry and IOS measurements in non-CAD and CAD patients.

	No CAD	n	CAD	n	P-value*	P-value**	P-value***
**VC**	3.79 (1.08)	237	3.71 (0.92)	159	**0.004**	**0.016**	N/A
**FEV**_**1**_	2.74 (0.87)	237	2.69 (0.80)	159	0.075	0.188	N/A
**D**_**LCO**_	7.17 (2.08)	237	6.64 (1.95)	158	**0.001**	**0.004**	**0.009**
**R 5Hz**	0.36 (0.13)	237	0.37 (0.14)	157	**0.026**	**0.036**	N/A
**R 20Hz**	0.30 (0.08)	237	0.29 (0.08)	157	0.172	0.229	N/A
**R 5Hz-20Hz**	0.06 (0.07)	227	0.07 (0.07)	153	0.073	0.090	N/A
**AX**	0.48 (0.84)	236	0.59 (0.98)	157	**0.005**	**0.011**	N/A

Displayed as mean (SD). P-values from linear regression analyses.

* Minimally adjusted model (age, sex, height, smoking, diabetes, hypertension).

** Adjusted model 1 (Minimally adjusted model + BNP).

*** Adjusted model 2 (Minimally adjusted model + BNP + FEV_1_).

CAD = Coronary artery disease, please see text for definition; R = Resistance; AX = Area of reactance.

VC (litres), FEV_1_ (litres), D_LCO_ (mmol/(min*kPa*l)), R 5Hz (kPa/(l/s)), R 20Hz (kPa/(l/s)), R 5Hz-20Hz (kPa/(l/s)), AX (kPa/l).

Both a heart failure diagnosis retrieved from the questionnaire (p = 0.004), and relative BNP (p = 0.001) were associated with decreased D_LCO_ by 1.181 and 0.655 mmol/(min*kPa*l), respectively.

There were no significant differences in lung function parameters when comparing the groups coronary ischemia versus no ischemia on MPI (Supplementary Table 1 in S1 Data).

### Protein biomarkers

Of the 92 analyzed cardiovascular proteomic biomarkers, a total of 30 proteins were significantly altered in patients with CAD compared with subjects without CAD (Table 3 and Supplementary Table 2a in S1 Data).

**Table 3:** Proteins significantly altered in subjects with CAD compared with subjects without CAD.

Protein	B	P-value adjusted*	Protein	B	P-value adjusted*
**VSIG2**	0.436	3.38E-07	**KIM1**	0.426	4.92E-03
**PRSS8**	0.274	5.10E-06	**IL-4RA**	0.192	5.56E-03
**REN**	0.642	9.32E-06	**MMP7**	0.211	5.68E-03
**Gal-9**	0.195	1.84E-05	**ADM**	0.221	6.74E-03
**IL-1ra**	0.323	1.84E-05	**XCL1**	0.193	7.63E-03
**TRAIL-R2**	0.332	3.07E-05	**BNP**	0.776	7.63E-03
**GIF**	0.462	3.94E-05	**IL18**	0.250	1.00E-02
**TNFRSF10A**	0.239	1.33E-04	**ACE2**	0.322	1.21E-02
**SPON2**	0.101	1.33E-04	**CCL3**	0.250	1.78E-02
**FABP2**	0.455	1.84E-04	**LPL**	-0.213	1.91E-02
**MMP12**	0.439	2.51E-04	**CTSL1**	0.168	2.11E-02
**TNFRSF11A**	0.285	1.08E-03	**IDUA**	0.131	2.11E-02
**LEP**	0.137	1.30E-03	**PSGL-1**	0.048	2.24E-02
**AMBP**	0.096	4.48E-03	**FGF-23**	0.276	3.75E-02
**GT**	0.288	4.48E-03	**TF**	0.101	4.79E-02

*Adjusted for age, sex, current smoking (dichotomous), and for multiple testing using Benjamini-Hochberg.

VSIG2 = V-set and immunoglobulin domain-containing protein 2; PRSS8 = Prostasin; REN = Renin; Gal-9 = Galectin-9; IL-1ra = Interleukin-1 receptor antagonist protein; TRAIL-R2 = TNF-related apoptosis-inducing ligand receptor 2; GIF = Gastric intrinsic factor; TNFRSF10A = Tumor necrosis factor receptor superfamily member 10A; SPON2 = Spondin-2; FABP2 = Fatty acid-binding protein, intestinal; MMP12 = Matrix metalloproteinase-12; TNFRSF11A = Tumor necrosis factor receptor superfamily member 11A; LEP = Leptin; AMBP = Protein AMBP; GT = Gastrotropin; KIM1 = Kidney Injury Molecule; IL-4RA = Interleukin-4 receptor subunit alpha; MMP7 = Matrix metalloproteinase-7; ADM = adrenomedullin; XCL1 = Lymphotactin; BNP = Brain natriuretic peptide; IL18 = Interleukin-18; ACE2 = Angiotensin-converting enzyme 2; CCL3 = C-C motif chemokine 3; LPL = Lipoprotein lipase; CTSL1 = Cathepsin L1; IDUA = Alpha-L-iduronidase; PSGL-1 = P-selectin glycoprotein ligand 1; FGF-23 = Fibroblast growth factor 23; TF = Tissue factor.

There were no significant differences in concentrations of the protein biomarkers between patients with and without airflow obstruction, accounting for multiple testing. (Supplementary Table 2b in S1 Data).

**Fig 3:**
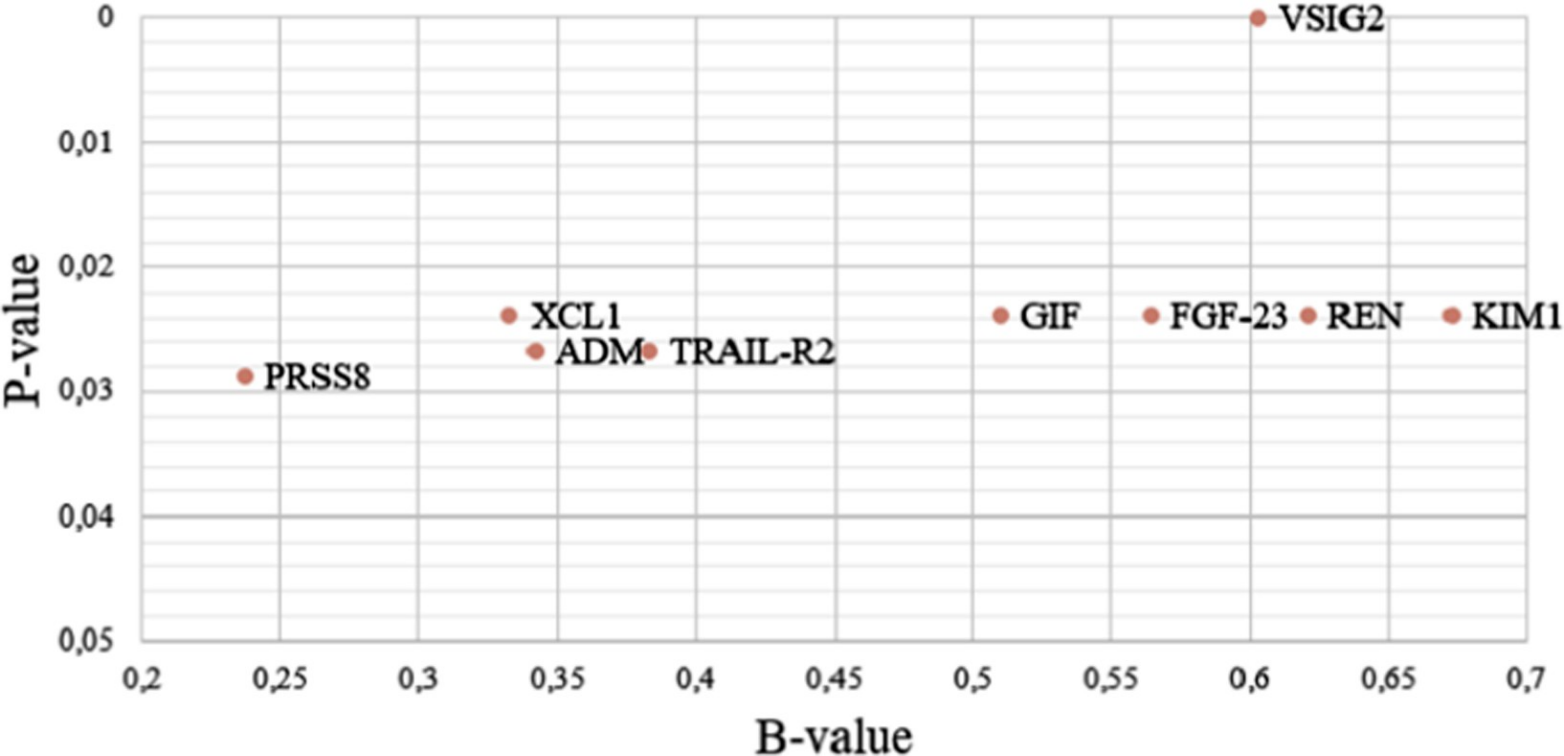
B-value vs P-value in 9 proteins significantly altered in subjects with combined diagnosis compared with no combined diagnosis. Legend: See Table 3 legend for protein definitions.

A total of nine proteins were significantly increased in subjects with a combined diagnosis of airflow obstruction and CAD compared with those without a combined diagnosis: *VSIG2*, *Kidney Injury Molecule (KIM1)*, *Fibroblast growth factor 23 (FGF-23)*, *Renin (REN)*, *Lymphotactin (XCL1)*, *Gastric intrinsic factor (GIF)*, *ADM*, *TNF-related apoptosis-inducing ligand receptor 2 (TRAIL-R2)* and *Prostasin (PRSS8)* (Fig 3 and Table 4, Supplementary Table 2c in S1 Data). All of these proteins were associated with isolated CAD, as well. Patients with a combined diagnosis had equally severe airflow obstruction as patients with isolated airflow obstruction, according to FEV_1_ (2.2 vs 2.3 L; p = 0.304).

**Table 4:** Proteins significantly altered in subjects with a combined diagnosis compared with no combined diagnosis.

Protein	B	P-value adjusted*	Protein	B	P-value adjusted*
**VSIG2**	0.603	4.36E-05	**GIF**	0.510	2.39E-02
**KIM1**	0.673	2.39E-02	**ADM**	0.342	2.68E-02
**FGF-23**	0.564	2.39E-02	**TRAIL-R2**	0.383	2.68E-02
**REN**	0.621	2.39E-02	**PRSS8**	0.237	2.88E-02
**XCL1**	0.333	2.39E-02			

*Adjusted for age, sex, current smoking (dichotomous), and for multiple testing using Benjamini-Hochberg Combined diagnosis = Diagnosis of CAD and airflow obstruction, please see text for definitions See Table 3 legend for protein definitions.

Among subjects with CAD, two proteins were significantly increased in subjects with ischemia on MPI compared to those without ischemia on MPI: *VSIG2* and *GIF* (Supplementary Table 2d in S1 Data).

## Discussion

In this explorative study, we aimed to identify common physiological and proteomic biomarkers of chronic airflow obstruction and stable coronary artery disease. We showed that subjects with CAD have a significantly lower diffusing capacity (D_LCO_) and a higher total airway resistance compared with subjects without CAD. Nine novel cardiovascular biomarkers were associated with a combined diagnosis of CAD and airflow obstruction, but no protein was associated with isolated airflow obstruction. Our findings are in line with the results of previous studies [27] that have shown a relationship between CVD (denoted as atherosclerosis in internal carotid artery) and decreased D_LCO_, but to our knowledge this study is the first to show an association between CAD and reduced D_LCO_.

It is well known that certain conditions that are not primarily related to the lung status can affect D_LCO_. Heart failure has been previously associated with decreased D_LCO_ [28], which was also the case in our current study. Furthermore, type II diabetes has been associated with a decrease in D_LCO_ in earlier studies [51]. Patients with CAD more often have heart failure (as a consequence) and diabetes (as a risk factor), which may explain the relation between CAD and D_LCO_ in our current study. However, the association between a decreased CAD and D_LCO_ remained significant after adjustments for BNP, a sensitive marker for heart failure, diabetes as well as other common risk factors for both CAD and airflow obstruction.

As briefly mentioned, a previous population-based study found an association between carotid plaques and low D_LCO_, also after adjusting for C-reactive protein and traditional atherosclerosis risk factors [27]. In the same study there was no significant association between plaque and FEV_1_ or COPD status. Our current study results are in line with these previous findings, i.e. the association between reduced D_LCO_ and CAD remained significant also after adjusting for FEV_1_. Thus, the lower D_LCO_ in subjects with CAD may not solely be explained by reduced spirometry values, meaning that the association between atherosclerosis and reduced lung function is unlikely to be explained only by bronchial obstruction and low-grade systemic inflammation. As already mentioned potential concurrent damage to elastin in the artery and the alveoli [12–15] as well as potential endothelial dysfunction in the coronary and pulmonary vasculature [18] may be one link explaining our results. An additional explanation for the relationship between D_LCO_ and CAD may be based on West, who has shown that increased pulmonary capillary pressure leads to rapid remodelling of the alveolar-capillary membrane [52]. During remodelling, the thickness of the membrane is increased in order to withstand a higher pressure, which leads to reduced diffusion across the membrane. Patients with stable CAD have a higher pressure in the pulmonary circulation during exertion [53]. Thus, numerous, brief episodes of increased capillary pressure in subjects with CAD may lead to a permanently increased thickness of the membrane and may explain part of the relationship between CAD and decreased D_LCO_.

In addition to lower D_LCO,_ subjects with CAD had higher total airway resistance (i.e. 5 Hz) in our current study, whereas there was no association between CAD and central airway resistance (i.e. 20 Hz). Similar to the association with D_LCO_ and CAD, the relationship between total airway resistance and CAD was significant after adjustment for common risk factors for airflow obstruction and CAD, as well as after adjustment for FEV_1_. In all, our results of reduced D_LCO_ and increased total airway resistance in patients with CAD, irrespective of decreased FEV_1_, may indicate a common mechanism that impairs the function of the airways and coronary arteries on top of the established association between reduced FEV_1_, VC and CAD.

The secondary aim of the current study was to identify novel cardiovascular plasma-biomarkers that associate with both CAD and airflow obstruction.

A total of 30 novel cardiovascular biomarkers were associated with CAD, which naturally is an expected result for this panel. On the contrary, no proteins were significantly associated solely with airflow obstruction. Moreover, even though nine biomarkers were increased in patients with a combined diagnosis of airflow obstruction and CAD compared to patients without a combined diagnosis, the levels of those nine proteins were also increased in patients with CAD. Thus, the association between the nine biomarkers and a combined diagnosis of airflow obstruction and CAD, may be driven primarily by the association between the nine biomarkers and CAD.

Of the nine proteins that were associated with a combined diagnosis of CAD and airflow obstruction, eight had a stronger association (higher beta and lower p-value) with CAD only than with a combined diagnosis whereas one protein, FGF-23, was more strongly associated with a combined diagnosis than with CAD only. FGF-23 is a protein involved in phosphate and vitamin D metabolism. It acts on the kidneys to decrease reabsorption and increase excretion of phosphate, thus decreasing its serum concentration. Increased level of serum phosphate is closely correlated with extent of inflammation and vascular calcification [54]. FGF-23 has been identified as an independent marker of COPD and decreased FEV_1_ and D_LCO_ [55]. Decreased FGF-23 and hyperphosphatemia in mice have been shown to be associated with emphysema and vascular calcification, among other signs of ageing. [56]. We have no data on phosphate concentration in our study meaning that we were unable to assess the relation between FGF-23, phosphate levels, CAD and airway obstruction. However, the hypothesis that phosphate metabolism may be involved in both CAD and COPD may be tested in future research.

A related possible link between FGF-23, CAD and COPD is the vitamin-D metabolism. FGF-23 acts by supressing 1-alpha-hydroxylase, an enzyme responsible for hydroxylation of calcifediol to the active form of vitamin D, calcitriol [57]. The protein thus acts to decrease the activity of vitamin D, a steroid known to inhibit inflammation by regulating the production of inflammatory cytokines [58]. Whether or not Vitamin-D is implicated in both CAD and COPD, is an additional hypothesis that may be further tested based on our current results.

Other biomarkers that were associated with a combined diagnosis included *Renin (REN)*, *adrenomedullin (ADM)*, *Prostasin (PRSS8)*, *TNF-related apoptosis-inducing ligand receptor 2 (TRAIL-R2)*, *Lymphotactin (XCL1)*, *Kidney Injury Molecule (KIM1)*, *V-Set And Immunoglobulin Domain Containing 2 (VSIG2)*, *and Gastric intrinsic factor (GIF)*. Renin is a well-known component in the cardiovascular homeostasis and involved within the Renin-angiotensin-aldosterone system (RAAS). It acts in the first step of the RAAS-cascade by cleaving angiotensinogen into angiotensin I. Angiotensin I is then converted into Angiotensin II which exert several actions, including stimulation of aldosterone synthesis. Activation of the RAAS has been related to increased atherosclerosis [59], but also to inflammatory diseases of the lung [60]. The end-product of the RAAS pathway, Angiotensin II, binds to Angiotensin II Type 1 receptor (AT1R) or Angiotensin II Type 2 receptor (AT2R). Activated AT1R mediates pro-inflammatory and pro-hyperresponsive actions, which AT2R does not. COPD patients have been shown to have a five-fold increase in AT1R to AT2R ratio in their lungs. The increased ratio also correlated with reduced lung function [61].

Adrenomedullin is expressed in multiple cell types and exerts various actions, including vaso- and bronchodilation. It has a vascular and cardiac protective role and prevents against the formation of atherosclerosis [62]. Levels of ADM have been shown to be higher in patients with atherosclerosis [62,63] and COPD [64]. Plasma levels of ADM also positively correlate with levels of inflammatory markers such as CRP and IL-6, which could explain the relationship between ADM and both atherosclerosis and airflow obstruction/COPD [62]. It may also be that COPD is the cause of increased ADM, since one study indicated that ADM is up-regulated by hypoxia [65].

Prostasin is involved in activation of sodium channels in renal and bronchial epithelium. A study on rats showed an increased blood pressure after gene transfer of human prostasin [66]. Aldosterone levels were also increased in the same study, suggesting that prostasin increases blood pressure by regulation of the RAAS. Its actions in the airway epithelium has mainly been related to cystic fibrosis. The authors found no earlier described correlation between PRSS8 and airflow obstruction in the literature.

TRAIL-R2, also known as Death receptor 5, is a plasma membrane receptor that is mainly known for regulating apoptosis [67,68]. It is a member of the TNF protein superfamily and is expressed by endothelial cells and vascular smooth muscle cells [69], where it has been shown to induce vascular inflammation. TRAIL-R2’s ligand TRAIL has been linked to vascular smooth muscle cells proliferation, promoting the formation and stabilization of atherosclerotic plaques [70]. High levels of soluble TRAIL-R2 can predict future cardiovascular events in the population [69]. Studies have linked TRAIL-R2 and its ligand to alveolar cell apoptosis in emphysema. Alveolar cells of an emphysematous lung have increased sensitivity to TRAIL mediates apoptosis [71] and have higher level of TRAIL receptors [72].

Lymphotactin (XCL1) is a cytokine involved in inflammatory response, thus fitting well into the hypothesis that inflammation is common denominator for CAD and COPD [41,73,74]. It has chemotactic properties towards CD8+ T cells and NK cells, while inhibiting proliferation of CD4+ T cells. One earlier study found increased XCL1 and decreased CD4+/CD8+ T cell ratio in lung tissue of COPD mice compared to control mice [75]. They also found that treating mice with exogenous XCL1 further decreased CD4+/CD8+ ratio. Decreased CD4+/CD8+ ratio is characteristic for COPD, which may be due to overexpression of XCL1 [75]. No relationship between XCL1 and CAD was found in the literature.

KIM1 protects the kidney from acute injury by down-regulating inflammation and mediating phagocytosis of apoptotic cellular debris [76]. KIM1 is up-regulated in various primary and secondary kidney diseases, and can be used as a marker for renal proximal tubule damage [77]. Increased levels of urinary KIM1 has been independently associated with higher risk of heart failure, cardiovascular events and death in patients with chronic kidney disease [78]. No studies relating KIM1 to COPD were found in the literature.

The authors found no studies relating VSIG2 and GIF to CAD, airflow obstruction and/or COPD in the published literature.

As a concluding remark from the overall negative proteomic analysis provided in this study, the authors think that the individual proteins may one by one associate with very small differences in the lung function parameters. Accordingly, a larger study sample may be required in order to be able to show potential differences in lung function parameters between subjects with different concentrations of the individual biomarkers. Moreover, the difference in relative concentrations that were used in the current study may be too small to be of importance for the phenotype, especially since the patients investigated were relatively homogenous group of high-risk patients. Naturally, none of the proteins investigated here could be used clinically, at the moment. Future research should be conducted in larger and less homogenous study samples, and if possible with absolute measurements, meaning that there may be a greater differences between individuals with different phenotypes.

### Limitations

The current study has a number of important limitations.

The study consists of cross-sectional analyses and as such, no assessments can be drawn about the temporal relationships among the variables. Similarly, no causal conclusions can be drawn.

The study was based in a cohort of patients presenting with symptoms suggestive of myocardial ischemia, and as such, the results cannot be generalized to the general population.

A total of 41% of those invited to the study declined to participate. This increases the risk of selection bias mainly of the type “healthy cohort effect”, which is well known in medical studies. We were not able to receive data for the subjects who declined to participate, due to ethical regulations.

We did not measure C-reactive protein in our subjects.

As previously mentioned, heart failure is associated with a decreased D_LCO_. We used relative BNP levels within the cohort to adjust for heart failure instead of the ejection fraction measured on MPI, because of its low accuracy. Using ejection fraction from MPI would also entail a risk of overlooking patients with diastolic heart failure. Ideally, all study participants would undergo echocardiography to assess heart failure and pulmonary hypertension.

Hemoglobin levels alter D_LCO_, with anemia causing it to decrease and polycythemia leading to an increased value, even though the impact of hemoglobin level on D_LCO_ has been found to be modest [79]. We did not have data on our patients Hb. However, it can be argued that hemoglobin levels should be approximately the same in patients with CAD as in patients without CAD.

Analyses on proteomic biomarkers were not performed on absolute values of the proteins, but on their relative value within the study population. Even though our study was of explorative nature, the use of relative instead of absolute levels adds some uncertainty and makes it harder to repeat the results in an unrelated cohort. Moreover, high number of analyses performed warrants adjustment for multiple testing. Such adjustments may also lead to over-adjustments, increasing the risk of a type II error.

Finally, unmeasured potential confounders, such as short-term air pollution exposure and subclinical disease with impact on both the coronary and respiratory systems, may have affected the results.

## Conclusions

Diffusing capacity for carbon monoxide is decreased in patients with CAD, indicating that patients with CAD have reduced gas exchange independently of diabetes, heart failure and decreased FEV_1_. Several cardiovascular biomarkers are associated with co-existent CAD and airflow obstruction, but none with airflow obstruction only. The current findings support the hypothesis that the relationship between reduced lung function, airflow obstruction and CAD is complex and involves other pathophysiological mechanisms than reduced ventilation only.

## Supporting information

S1 Data (DOCX)
