# Supplementary material for: Common physiologic and proteomic biomarkers in pulmonary and coronary artery disease
Source: PLoS One. 2022 Mar 9;17(3):e0264376. doi: 10.1371/journal.pone.0264376 (PMC8906634; doi:10.1371/journal.pone.0264376)
Supplement: S1 Data — (DOCX) [file pone.0264376.s001.docx]

**Supplementary Table 1**

| **Supplementary Table 1** | | | |
| --- | --- | --- | --- |
| **Association between coronary ischemia and measurements of spirometry and IOS** | | | |
|  | B | P-value* | P-value** |
| VC | -0.050 | 0.340 | 0.533 |
| FEV_1_ | 0.024 | 0.986 | 0.751 |
| D_LCO_ | -0.172 | 0.179 | 0.369 |
| R 5 Hz | 0.018 | 0.287 | 0.330 |
| R 20 Hz | 0.004 | 0.453 | 0.520 |
| R 5 Hz – 20 Hz | 0.014 | 0.173 | 0.197 |
| AX | 0.146 | 0.184 | 0.250 |

* Minimally adjusted model (age, sex, height, smoking, diabetes, hypertension)
** Adjusted model 1 (Minimally adjusted model + BNP)
Ischemia was assessed by myocardial perfusion imaging
IOS = Impulse oscillometry
VC = Vital capacity (litres); FEV_1_ = Forced expiratory volume in one second (litres); D_LCO_ = Diffusing capacity for carbon monoxide (mmol/(min*kPa*l)); R = Resistance (kPa/(l/s)); AX= Area of reactance (kPa/l)

**Protein definitions:**

| ACE2 = Angiotensin-converting enzyme 2 |
| --- |
| ADAM-TS13 = A disintegrin and metalloproteinase with thrombospondin motifs 13 |
| ADM = adrenomedullin |
| AGRP = Agouti-related protein |
| AMBP = Protein AMBP |
| ANG-1 = Angiopoietin-1 |
| BMP-6 = Bone morphogenetic protein 6 |
| BNP = Brain natriuretic peptide |
| BOC = Brother of CDO |
| CA5A = Carbonic anhydrase 5A, mitochondrial |
| CCL17 = C-C motif chemokine 17 |
| CCL3 = C-C motif chemokine 3 |
| CD4 = T-cell surface glycoprotein CD4 |
| CD40-L = CD40 ligand |
| CD84 = SLAM family member 5 |
| CEACAM8 = Carcinoembryonic antigenrelated cell adhesion molecule 8 |
| CTRC = Chymotrypsin C |
| CTSL1 = Cathepsin L1 |
| CXCL1 = C-X-C motif chemokine 1 |
| DCN = Decorin |
| DECR1 = 2,4-dienoyl-CoA reductase, mitochondrial |
| Dkk-1 = Dickkopf-related protein 1 |
| FABP2 = Fatty acid-binding protein, intestinal |
| FGF-21 = Fibroblast growth factor 21 |
| FGF-23 = Fibroblast growth factor 23 |
| FS = Follistatin |
| Gal-9 = Galectin-9 |
| GDF-2 = Growth/differentiation factor 2 |
| GH = Growth hormone |
| GIF = Gastric intrinsic factor |
| GLO1 = Lactoylglutathione lyase |
| GT = Gastrotropin |
| HAOX1 = Hydroxyacid oxidase 1 |
| HB-EGF = Proheparin-binding EGF-like growth factor |
| HO-1 = Heme oxygenase 1 |
| hOSCAR = Osteoclast-associated immunoglobulin-like receptor |
| HSP 27 = Heat shock 27 kDa protein |
| IDUA = Alpha-L-iduronidase |
| IgG Fc receptor II-b = Low affinity immunoglobulin gamma Fc region receptor II-b |
| IL16 = Pro-interleukin-16 |
| IL-17D = Interleukin-17D |
| IL18 = Interleukin-18 |
| IL-1ra = Interleukin-1 receptor antagonist protein |
| IL1RL2 = Interleukin-1 receptor-like 2 |
| IL-27 = Interleukin-27 |
| IL-4RA = Interleukin-4 receptor subunit alpha |
| IL6 = Interleukin-6 |
| ITGB1BP2 = Melusin |
| KIM1 = Kidney Injury Molecule |
| LEP = Leptin |
| LOX-1 = Lectin-like oxidized LDL receptor 1 |
| LPL = Lipoprotein lipase |
| MARCO = Macrophage receptor MARCO |
| MERTK = Tyrosine-protein kinase Mer |
| MMP12 = Matrix metalloproteinase-12 |
| MMP7 = Matrix metalloproteinase-7 |
| NEMO = NF-kappa-B essential modulator |
| PAPPA = Pappalysin-1 |
| PAR-1 = Proteinase-activated receptor 1 |
| PARP-1 = Poly [ADP-ribose] polymerase 1 |
| PDGF subunit B = Platelet-derived growth factor subunit B |
| PD-L2 = Programmed cell death 1 ligand 2 |
| PGF = Placenta growth factor |
| PIgR = Polymeric immunoglobulin receptor |
| PRELP = Prolargin |
| PRSS27 = Serine protease 27 |
| PRSS8 = Prostasin |
| PSGL-1 = P-selectin glycoprotein ligand 1 |
| PTX3 = Pentraxin-related protein PTX3 |
| RAGE = Receptor for advanced glycosylation end products |
| REN = Renin |
| SCF = Stem cell factor |
| SERPINA12 = Serpin A12 |
| SLAMF7 = SLAM family member 7 |
| SOD2 = Superoxide dismutase [Mn], mitochondrial |
| SORT1 = Sortilin |
| SPON2 = Spondin-2 |
| SRC = Proto-oncogene tyrosine-protein kinase Src |
| STK4 = Serine/threonine-protein kinase 4 |
| TF = Tissue factor |
| TGM2 = Protein-glutamine gamma-glutamyltransferase 2 |
| THBS2 = Thrombospondin-2 |
| THPO = Thrombopoietin |
| TIE2 = Angiopoietin-1 receptor |
| TM = Thrombomodulin |
| TNFRSF10A = Tumor necrosis factor receptor superfamily member 10A |
| TNFRSF11A = Tumor necrosis factor receptor superfamily member 11A |
| TNFRSF13B = Tumor necrosis factor receptor superfamily member 13B |
| TRAIL-R2 = TNF-related apoptosis-inducing ligand receptor 2 |
| VEGFD = Vascular endothelial growth factor D |
| VSIG2 = V-set and immunoglobulin domain-containing protein 2 |
| XCL1 = Lymphotactin |

**Supplementary Table 2**

**Supplementary table 2a. Significance of protein concentration difference in subjects with CAD, compared to no CAD**

| **Name** | **p-value** | **Adjusted p-value*** | **Threshold** |
| --- | --- | --- | --- |
| VSIG2 | 3.67E-09 | 3.38E-07 | Significant |
| PRSS8 | 1.11E-07 | 5.10E-06 | Significant |
| REN | 3.04E-07 | 9.32E-06 | Significant |
| Gal-9 | 1.00E-06 | 1.84E-05 | Significant |
| IL-1ra | 1.00E-06 | 1.84E-05 | Significant |
| TRAIL-R2 | 2.00E-06 | 3.07E-05 | Significant |
| GIF | 3.00E-06 | 3.94E-05 | Significant |
| TNFRSF10A | 1.20E-05 | 1.33E-04 | Significant |
| SPON2 | 1.30E-05 | 1.33E-04 | Significant |
| FABP2 | 2.00E-05 | 1.84E-04 | Significant |
| MMP12 | 3.00E-05 | 2.51E-04 | Significant |
| TNFRSF11A | 1.41E-04 | 1.08E-03 | Significant |
| LEP | 1.84E-04 | 1.30E-03 | Significant |
| AMBP | 6.85E-04 | 4.48E-03 | Significant |
| GT | 7.30E-04 | 4.48E-03 | Significant |
| KIM1 | 8.55E-04 | 4.92E-03 | Significant |
| IL-4RA | 1.03E-03 | 5.56E-03 | Significant |
| MMP7 | 1.11E-03 | 5.68E-03 | Significant |
| ADM | 1.39E-03 | 6.74E-03 | Significant |
| XCL1 | 1.72E-03 | 7.63E-03 | Significant |
| BNP | 1.74E-03 | 7.63E-03 | Significant |
| IL18 | 2.40E-03 | 1.00E-02 | Significant |
| ACE2 | 3.03E-03 | 1.21E-02 | Significant |
| CCL3 | 4.65E-03 | 1.78E-02 | Significant |
| LPL | 5.20E-03 | 1.91E-02 | Significant |
| CTSL1 | 6.18E-03 | 2.11E-02 | Significant |
| IDUA | 6.19E-03 | 2.11E-02 | Significant |
| PSGL-1 | 6.82E-03 | 2.24E-02 | Significant |
| FGF-23 | 1.18E-02 | 3.75E-02 | Significant |
| TF | 1.56E-02 | 4.79E-02 | Significant |
| FGF-21 | 2.52E-02 | 7.49E-02 | Non-Significant |
| CD4 | 3.05E-02 | 8.76E-02 | Non-Significant |
| SOD2 | 3.28E-02 | 8.92E-02 | Non-Significant |
| IL6 | 3.30E-02 | 8.92E-02 | Non-Significant |
| hOSCAR | 3.60E-02 | 9.46E-02 | Non-Significant |
| PARP-1 | 4.40E-02 | 1.12E-01 | Non-Significant |
| PGF | 4.54E-02 | 1.13E-01 | Non-Significant |
| TNFRSF13B | 5.08E-02 | 1.23E-01 | Non-Significant |
| HO-1 | 5.66E-02 | 1.34E-01 | Non-Significant |
| PAPPA | 6.66E-02 | 1.53E-01 | Non-Significant |
| THBS2 | 6.83E-02 | 1.53E-01 | Non-Significant |
| PD-L2 | 1.05E-01 | 2.30E-01 | Non-Significant |
| SORT1 | 1.14E-01 | 2.43E-01 | Non-Significant |
| THPO | 1.22E-01 | 2.54E-01 | Non-Significant |
| TM | 1.37E-01 | 2.80E-01 | Non-Significant |
| CA5A | 1.54E-01 | 3.02E-01 | Non-Significant |
| MERTK | 1.54E-01 | 3.02E-01 | Non-Significant |
| FS | 1.69E-01 | 3.21E-01 | Non-Significant |
| PAR-1 | 1.71E-01 | 3.21E-01 | Non-Significant |
| IL16 | 1.75E-01 | 3.22E-01 | Non-Significant |
| NEMO | 1.90E-01 | 3.43E-01 | Non-Significant |
| CD40-L | 1.99E-01 | 3.52E-01 | Non-Significant |
| ITGB1BP2 | 2.09E-01 | 3.63E-01 | Non-Significant |
| SERPINA12 | 2.24E-01 | 3.82E-01 | Non-Significant |
| STK4 | 2.39E-01 | 4.00E-01 | Non-Significant |
| HAOX1 | 2.51E-01 | 4.12E-01 | Non-Significant |
| GLO1 | 2.77E-01 | 4.44E-01 | Non-Significant |
| SCF | 2.80E-01 | 4.44E-01 | Non-Significant |
| LOX-1 | 2.87E-01 | 4.45E-01 | Non-Significant |
| Dkk-1 | 2.90E-01 | 4.45E-01 | Non-Significant |
| CCL17 | 3.07E-01 | 4.57E-01 | Non-Significant |
| SLAMF7 | 3.08E-01 | 4.57E-01 | Non-Significant |
| CD84 | 3.15E-01 | 4.59E-01 | Non-Significant |
| DCN | 3.29E-01 | 4.72E-01 | Non-Significant |
| PRELP | 3.37E-01 | 4.77E-01 | Non-Significant |
| RAGE | 4.10E-01 | 5.72E-01 | Non-Significant |
| IL1RL2 | 4.25E-01 | 5.83E-01 | Non-Significant |
| AGRP | 4.32E-01 | 5.85E-01 | Non-Significant |
| MARCO | 4.47E-01 | 5.97E-01 | Non-Significant |
| DECR1 | 4.78E-01 | 6.23E-01 | Non-Significant |
| PIgR | 4.81E-01 | 6.23E-01 | Non-Significant |
| CXCL1 | 6.02E-01 | 7.64E-01 | Non-Significant |
| SRC | 6.06E-01 | 7.64E-01 | Non-Significant |
| PRSS27 | 6.36E-01 | 7.86E-01 | Non-Significant |
| ADAM-TS13 | 6.41E-01 | 7.86E-01 | Non-Significant |
| HB-EGF | 6.70E-01 | 8.04E-01 | Non-Significant |
| TGM2 | 6.80E-01 | 8.04E-01 | Non-Significant |
| PTX3 | 6.82E-01 | 8.04E-01 | Non-Significant |
| VEGFD | 7.24E-01 | 8.31E-01 | Non-Significant |
| GDF-2 | 7.33E-01 | 8.31E-01 | Non-Significant |
| ANG-1 | 7.36E-01 | 8.31E-01 | Non-Significant |
| IL-27 | 7.41E-01 | 8.31E-01 | Non-Significant |
| TIE2 | 7.61E-01 | 8.43E-01 | Non-Significant |
| GH | 7.78E-01 | 8.44E-01 | Non-Significant |
| CEACAM8 | 7.80E-01 | 8.44E-01 | Non-Significant |
| IL-17D | 7.91E-01 | 8.47E-01 | Non-Significant |
| BMP-6 | 8.48E-01 | 8.97E-01 | Non-Significant |
| CTRC | 8.93E-01 | 9.34E-01 | Non-Significant |
| BOC | 9.22E-01 | 9.53E-01 | Non-Significant |
| PDGF subunit B | 9.39E-01 | 9.57E-01 | Non-Significant |
| IgG Fc receptor II-b | 9.47E-01 | 9.57E-01 | Non-Significant |
| HSP 27 | 1.00E+00 | 1.00E+00 | Non-Significant |

*Adjusted for mass significance using Benjamini-Hochberg method.
Threshold = Adjusted p-value < 0.05

**Supplementary table 2b. Significance of protein concentration difference in subjects with airflow obstruction, compared to no airflow obstruction.**

| **Name** | **p-value** | **Adjusted p-value** | **Threshold** |
| --- | --- | --- | --- |
| ADM | 7.64E-03 | 3.95E-01 | Non-significant |
| VSIG2 | 8.59E-03 | 3.95E-01 | Non-significant |
| FS | 1.32E-02 | 4.06E-01 | Non-significant |
| CEACAM8 | 2.28E-02 | 5.25E-01 | Non-significant |
| SOD2 | 7.87E-02 | 6.53E-01 | Non-significant |
| FGF-23 | 7.26E-02 | 6.53E-01 | Non-significant |
| REN | 6.87E-02 | 6.53E-01 | Non-significant |
| KIM1 | 7.55E-02 | 6.53E-01 | Non-significant |
| XCL1 | 8.52E-02 | 6.53E-01 | Non-significant |
| DCN | 7.60E-02 | 6.53E-01 | Non-significant |
| ACE2 | 6.90E-02 | 6.53E-01 | Non-significant |
| LEP | 7.85E-02 | 6.53E-01 | Non-significant |
| BMP-6 | 1.51E-01 | 6.79E-01 | Non-significant |
| SLAMF7 | 2.91E-01 | 6.79E-01 | Non-significant |
| PGF | 2.06E-01 | 6.79E-01 | Non-significant |
| ADAM-TS13 | 3.18E-01 | 6.79E-01 | Non-significant |
| BOC | 3.25E-01 | 6.79E-01 | Non-significant |
| IL-4RA | 2.17E-01 | 6.79E-01 | Non-significant |
| SRC | 1.92E-01 | 6.79E-01 | Non-significant |
| IL6 | 1.64E-01 | 6.79E-01 | Non-significant |
| TNFRSF10A | 1.23E-01 | 6.79E-01 | Non-significant |
| PAR-1 | 2.65E-01 | 6.79E-01 | Non-significant |
| TRAIL-R2 | 2.54E-01 | 6.79E-01 | Non-significant |
| TF | 3.10E-01 | 6.79E-01 | Non-significant |
| IL-17D | 1.63E-01 | 6.79E-01 | Non-significant |
| GIF | 2.32E-01 | 6.79E-01 | Non-significant |
| FGF-21 | 2.23E-01 | 6.79E-01 | Non-significant |
| PIgR | 3.07E-01 | 6.79E-01 | Non-significant |
| SPON2 | 1.19E-01 | 6.79E-01 | Non-significant |
| GH | 3.20E-01 | 6.79E-01 | Non-significant |
| CD84 | 2.80E-01 | 6.79E-01 | Non-significant |
| PAPPA | 1.54E-01 | 6.79E-01 | Non-significant |
| AMBP | 1.23E-01 | 6.79E-01 | Non-significant |
| PSGL-1 | 2.54E-01 | 6.79E-01 | Non-significant |
| MMP7 | 2.30E-01 | 6.79E-01 | Non-significant |
| IgG Fc receptor II-b | 1.82E-01 | 6.79E-01 | Non-significant |
| PRSS8 | 2.51E-01 | 6.79E-01 | Non-significant |
| AGRP | 3.19E-01 | 6.79E-01 | Non-significant |
| GDF-2 | 2.05E-01 | 6.79E-01 | Non-significant |
| TGM2 | 3.11E-01 | 6.79E-01 | Non-significant |
| CA5A | 2.26E-01 | 6.79E-01 | Non-significant |
| CD4 | 1.36E-01 | 6.79E-01 | Non-significant |
| PARP-1 | 2.28E-01 | 6.79E-01 | Non-significant |
| HAOX1 | 1.81E-01 | 6.79E-01 | Non-significant |
| hOSCAR | 3.34E-01 | 6.84E-01 | Non-significant |
| IL-1ra | 3.58E-01 | 7.00E-01 | Non-significant |
| ITGB1BP2 | 3.68E-01 | 7.00E-01 | Non-significant |
| PD-L2 | 3.56E-01 | 7.00E-01 | Non-significant |
| HSP 27 | 3.73E-01 | 7.00E-01 | Non-significant |
| HB-EGF | 3.82E-01 | 7.03E-01 | Non-significant |
| THBS2 | 4.00E-01 | 7.22E-01 | Non-significant |
| PDGF subunit B | 4.32E-01 | 7.64E-01 | Non-significant |
| SCF | 4.75E-01 | 7.67E-01 | Non-significant |
| PTX3 | 4.71E-01 | 7.67E-01 | Non-significant |
| CCL17 | 4.44E-01 | 7.67E-01 | Non-significant |
| GT | 4.75E-01 | 7.67E-01 | Non-significant |
| CTSL1 | 4.58E-01 | 7.67E-01 | Non-significant |
| TNFRSF11A | 4.92E-01 | 7.81E-01 | Non-significant |
| GLO1 | 5.21E-01 | 7.99E-01 | Non-significant |
| SORT1 | 5.18E-01 | 7.99E-01 | Non-significant |
| CXCL1 | 5.85E-01 | 8.17E-01 | Non-significant |
| Gal-9 | 5.66E-01 | 8.17E-01 | Non-significant |
| CTRC | 5.86E-01 | 8.17E-01 | Non-significant |
| PRELP | 5.80E-01 | 8.17E-01 | Non-significant |
| CCL3 | 5.77E-01 | 8.17E-01 | Non-significant |
| MARCO | 5.71E-01 | 8.17E-01 | Non-significant |
| MMP12 | 6.14E-01 | 8.43E-01 | Non-significant |
| TM | 6.64E-01 | 8.99E-01 | Non-significant |
| STK4 | 7.37E-01 | 9.36E-01 | Non-significant |
| LOX-1 | 7.53E-01 | 9.36E-01 | Non-significant |
| MERTK | 7.17E-01 | 9.36E-01 | Non-significant |
| IL16 | 7.48E-01 | 9.36E-01 | Non-significant |
| Dkk-1 | 7.39E-01 | 9.36E-01 | Non-significant |
| TNFRSF13B | 7.06E-01 | 9.36E-01 | Non-significant |
| IDUA | 7.91E-01 | 9.56E-01 | Non-significant |
| PRSS27 | 7.98E-01 | 9.56E-01 | Non-significant |
| IL18 | 8.00E-01 | 9.56E-01 | Non-significant |
| TIE2 | 8.11E-01 | 9.56E-01 | Non-significant |
| IL1RL2 | 8.36E-01 | 9.66E-01 | Non-significant |
| DECR1 | 8.40E-01 | 9.66E-01 | Non-significant |
| CD40-L | 9.00E-01 | 9.74E-01 | Non-significant |
| SERPINA12 | 9.00E-01 | 9.74E-01 | Non-significant |
| LPL | 8.90E-01 | 9.74E-01 | Non-significant |
| FABP2 | 8.93E-01 | 9.74E-01 | Non-significant |
| THPO | 8.96E-01 | 9.74E-01 | Non-significant |
| ANG-1 | 9.20E-01 | 9.79E-01 | Non-significant |
| IL-27 | 9.26E-01 | 9.79E-01 | Non-significant |
| HO-1 | 9.38E-01 | 9.80E-01 | Non-significant |
| RAGE | 9.51E-01 | 9.83E-01 | Non-significant |
| BNP | 9.97E-01 | 9.97E-01 | Non-significant |
| NEMO | 9.95E-01 | 9.97E-01 | Non-significant |
| VEGFD | 9.87E-01 | 9.97E-01 | Non-significant |

*Adjusted for mass significance using Benjamini-Hochberg method.
Threshold = Adjusted p-value < 0.05

**Supplementary table 2c. Significance of protein concentration difference in subjects with combined diagnosis, compared to no combined diagnosis**

| **Name** | **p-value** | **Adjusted p-value** | **Threshold** |
| --- | --- | --- | --- |
| VSIG2 | 4.73E-07 | 4.36E-05 | Significant |
| KIM1 | 6.19E-04 | 2.39E-02 | Significant |
| FGF-23 | 8.99E-04 | 2.39E-02 | Significant |
| REN | 1.22E-03 | 2.39E-02 | Significant |
| XCL1 | 1.39E-03 | 2.39E-02 | Significant |
| GIF | 1.56E-03 | 2.39E-02 | Significant |
| ADM | 2.33E-03 | 2.68E-02 | Significant |
| TRAIL-R2 | 2.33E-03 | 2.68E-02 | Significant |
| PRSS8 | 2.82E-03 | 2.88E-02 | Significant |
| IL-4RA | 5.50E-03 | 5.06E-02 | Non-significant |
| LEP | 7.29E-03 | 5.60E-02 | Non-significant |
| AMBP | 7.30E-03 | 5.60E-02 | Non-significant |
| FABP2 | 8.73E-03 | 6.18E-02 | Non-significant |
| SOD2 | 1.16E-02 | 7.14E-02 | Non-significant |
| Gal-9 | 1.16E-02 | 7.14E-02 | Non-significant |
| ACE2 | 1.33E-02 | 7.48E-02 | Non-significant |
| SPON2 | 1.38E-02 | 7.48E-02 | Non-significant |
| GT | 1.56E-02 | 7.95E-02 | Non-significant |
| MMP12 | 1.73E-02 | 8.40E-02 | Non-significant |
| MMP7 | 1.83E-02 | 8.42E-02 | Non-significant |
| TNFRSF11A | 2.07E-02 | 9.08E-02 | Non-significant |
| PSGL-1 | 2.68E-02 | 1.12E-01 | Non-significant |
| IL-1ra | 3.13E-02 | 1.25E-01 | Non-significant |
| HAOX1 | 4.86E-02 | 1.86E-01 | Non-significant |
| TNFRSF10A | 5.78E-02 | 2.06E-01 | Non-significant |
| PIgR | 5.82E-02 | 2.06E-01 | Non-significant |
| PD-L2 | 7.55E-02 | 2.57E-01 | Non-significant |
| CA5A | 8.21E-02 | 2.70E-01 | Non-significant |
| GDF-2 | 9.51E-02 | 3.02E-01 | Non-significant |
| THBS2 | 1.03E-01 | 3.15E-01 | Non-significant |
| CD4 | 1.11E-01 | 3.28E-01 | Non-significant |
| IL6 | 1.15E-01 | 3.31E-01 | Non-significant |
| CTSL1 | 1.44E-01 | 3.86E-01 | Non-significant |
| PGF | 1.47E-01 | 3.86E-01 | Non-significant |
| HO-1 | 1.54E-01 | 3.86E-01 | Non-significant |
| SERPINA12 | 1.54E-01 | 3.86E-01 | Non-significant |
| hOSCAR | 1.55E-01 | 3.86E-01 | Non-significant |
| CEACAM8 | 1.63E-01 | 3.94E-01 | Non-significant |
| FGF-21 | 1.87E-01 | 4.28E-01 | Non-significant |
| PAR-1 | 1.91E-01 | 4.28E-01 | Non-significant |
| CTRC | 1.93E-01 | 4.28E-01 | Non-significant |
| CCL17 | 1.95E-01 | 4.28E-01 | Non-significant |
| GLO1 | 2.00E-01 | 4.28E-01 | Non-significant |
| BNP | 2.14E-01 | 4.37E-01 | Non-significant |
| TF | 2.14E-01 | 4.37E-01 | Non-significant |
| IL-17D | 2.25E-01 | 4.42E-01 | Non-significant |
| CCL3 | 2.30E-01 | 4.42E-01 | Non-significant |
| ADAM-TS13 | 2.30E-01 | 4.42E-01 | Non-significant |
| MERTK | 2.57E-01 | 4.74E-01 | Non-significant |
| PARP-1 | 2.58E-01 | 4.74E-01 | Non-significant |
| TNFRSF13B | 2.75E-01 | 4.86E-01 | Non-significant |
| BOC | 2.75E-01 | 4.86E-01 | Non-significant |
| GH | 3.27E-01 | 5.68E-01 | Non-significant |
| PTX3 | 3.39E-01 | 5.78E-01 | Non-significant |
| CD84 | 3.50E-01 | 5.85E-01 | Non-significant |
| PRSS27 | 4.19E-01 | 6.74E-01 | Non-significant |
| IDUA | 4.26E-01 | 6.74E-01 | Non-significant |
| TM | 4.30E-01 | 6.74E-01 | Non-significant |
| IL1RL2 | 4.39E-01 | 6.74E-01 | Non-significant |
| VEGFD | 4.39E-01 | 6.74E-01 | Non-significant |
| SCF | 4.68E-01 | 7.06E-01 | Non-significant |
| AGRP | 4.86E-01 | 7.20E-01 | Non-significant |
| HB-EGF | 5.11E-01 | 7.46E-01 | Non-significant |
| RAGE | 5.35E-01 | 7.60E-01 | Non-significant |
| TGM2 | 5.37E-01 | 7.60E-01 | Non-significant |
| PAPPA | 5.46E-01 | 7.61E-01 | Non-significant |
| HSP 27 | 5.56E-01 | 7.64E-01 | Non-significant |
| ITGB1BP2 | 5.65E-01 | 7.65E-01 | Non-significant |
| LOX-1 | 6.22E-01 | 8.29E-01 | Non-significant |
| IL18 | 6.45E-01 | 8.29E-01 | Non-significant |
| THPO | 6.48E-01 | 8.29E-01 | Non-significant |
| SRC | 6.62E-01 | 8.29E-01 | Non-significant |
| DCN | 6.71E-01 | 8.29E-01 | Non-significant |
| IgG Fc receptor II-b | 6.79E-01 | 8.29E-01 | Non-significant |
| LPL | 6.84E-01 | 8.29E-01 | Non-significant |
| CD40-L | 7.03E-01 | 8.29E-01 | Non-significant |
| TIE2 | 7.05E-01 | 8.29E-01 | Non-significant |
| BMP-6 | 7.08E-01 | 8.29E-01 | Non-significant |
| NEMO | 7.20E-01 | 8.29E-01 | Non-significant |
| SORT1 | 7.22E-01 | 8.29E-01 | Non-significant |
| Dkk-1 | 7.30E-01 | 8.29E-01 | Non-significant |
| CXCL1 | 7.43E-01 | 8.34E-01 | Non-significant |
| IL-27 | 7.57E-01 | 8.39E-01 | Non-significant |
| IL16 | 7.70E-01 | 8.43E-01 | Non-significant |
| ANG-1 | 8.03E-01 | 8.66E-01 | Non-significant |
| SLAMF7 | 8.10E-01 | 8.66E-01 | Non-significant |
| PDGF subunit B | 8.19E-01 | 8.66E-01 | Non-significant |
| FS | 8.32E-01 | 8.68E-01 | Non-significant |
| PRELP | 8.39E-01 | 8.68E-01 | Non-significant |
| MARCO | 8.97E-01 | 9.10E-01 | Non-significant |
| STK4 | 9.10E-01 | 9.10E-01 | Non-significant |
| DECR1 | 9.10E-01 | 9.10E-01 | Non-significant |

*Adjusted for mass significance using Benjamini-Hochberg method.
Threshold = Adjusted p-value < 0.05

**Supplementary table 2d. Significance of protein concentration difference in subjects with ischemia on MPI, compared to no ischemia on MPI**

| **Name** | **p-value** | **Adjusted p-value** | **Threshold** |
| --- | --- | --- | --- |
| VSIG2 | 1.99E-04 | 1.83E-02 | Significant |
| GIF | 1.05E-03 | 4.84E-02 | Significant |
| REN | 1.49E-02 | 4.56E-01 | Non-significant |
| GT | 2.62E-02 | 6.03E-01 | Non-significant |
| CTRC | 4.63E-02 | 8.52E-01 | Non-significant |
| BMP-6 | 2.21E-01 | 8.82E-01 | Non-significant |
| ANG-1 | 2.47E-01 | 8.82E-01 | Non-significant |
| SLAMF7 | 1.30E-01 | 8.82E-01 | Non-significant |
| PGF | 3.90E-01 | 8.82E-01 | Non-significant |
| ADAM-TS13 | 2.63E-01 | 8.82E-01 | Non-significant |
| BOC | 2.68E-01 | 8.82E-01 | Non-significant |
| IL-4RA | 3.13E-01 | 8.82E-01 | Non-significant |
| SRC | 4.00E-01 | 8.82E-01 | Non-significant |
| IL-1ra | 3.61E-01 | 8.82E-01 | Non-significant |
| TNFRSF10A | 2.24E-01 | 8.82E-01 | Non-significant |
| STK4 | 3.21E-01 | 8.82E-01 | Non-significant |
| TNFRSF11A | 4.19E-01 | 8.82E-01 | Non-significant |
| PRSS27 | 3.87E-01 | 8.82E-01 | Non-significant |
| TIE2 | 3.56E-01 | 8.82E-01 | Non-significant |
| TF | 2.41E-01 | 8.82E-01 | Non-significant |
| IL1RL2 | 6.49E-02 | 8.82E-01 | Non-significant |
| IL-17D | 3.96E-01 | 8.82E-01 | Non-significant |
| LOX-1 | 3.56E-01 | 8.82E-01 | Non-significant |
| IL18 | 3.95E-01 | 8.82E-01 | Non-significant |
| PIgR | 1.27E-01 | 8.82E-01 | Non-significant |
| RAGE | 3.66E-01 | 8.82E-01 | Non-significant |
| SOD2 | 4.28E-01 | 8.82E-01 | Non-significant |
| FGF-23 | 2.14E-01 | 8.82E-01 | Non-significant |
| SPON2 | 4.51E-01 | 8.82E-01 | Non-significant |
| FS | 4.49E-01 | 8.82E-01 | Non-significant |
| GLO1 | 3.97E-01 | 8.82E-01 | Non-significant |
| THBS2 | 1.33E-01 | 8.82E-01 | Non-significant |
| TM | 4.28E-01 | 8.82E-01 | Non-significant |
| AMBP | 3.85E-01 | 8.82E-01 | Non-significant |
| PRELP | 2.05E-01 | 8.82E-01 | Non-significant |
| PTX3 | 2.46E-01 | 8.82E-01 | Non-significant |
| IgG Fc receptor II-b | 4.48E-01 | 8.82E-01 | Non-significant |
| DCN | 1.75E-01 | 8.82E-01 | Non-significant |
| PRSS8 | 3.41E-01 | 8.82E-01 | Non-significant |
| HB-EGF | 3.88E-01 | 8.82E-01 | Non-significant |
| FABP2 | 1.31E-01 | 8.82E-01 | Non-significant |
| BNP | 1.56E-01 | 8.82E-01 | Non-significant |
| ACE2 | 9.36E-02 | 8.82E-01 | Non-significant |
| hOSCAR | 3.46E-01 | 8.82E-01 | Non-significant |
| TNFRSF13B | 2.35E-01 | 8.82E-01 | Non-significant |
| LEP | 3.85E-01 | 8.82E-01 | Non-significant |
| VEGFD | 2.54E-01 | 8.82E-01 | Non-significant |
| TRAIL-R2 | 5.04E-01 | 8.83E-01 | Non-significant |
| SERPINA12 | 5.09E-01 | 8.83E-01 | Non-significant |
| IL16 | 5.02E-01 | 8.83E-01 | Non-significant |
| SORT1 | 4.93E-01 | 8.83E-01 | Non-significant |
| ITGB1BP2 | 5.07E-01 | 8.83E-01 | Non-significant |
| CA5A | 4.89E-01 | 8.83E-01 | Non-significant |
| CEACAM8 | 5.24E-01 | 8.92E-01 | Non-significant |
| CXCL1 | 5.83E-01 | 9.75E-01 | Non-significant |
| ADM | 8.01E-01 | 9.95E-01 | Non-significant |
| CD40-L | 9.84E-01 | 9.95E-01 | Non-significant |
| IL6 | 7.66E-01 | 9.95E-01 | Non-significant |
| IDUA | 7.20E-01 | 9.95E-01 | Non-significant |
| PAR-1 | 7.33E-01 | 9.95E-01 | Non-significant |
| PDGF subunit B | 6.85E-01 | 9.95E-01 | Non-significant |
| IL-27 | 7.95E-01 | 9.95E-01 | Non-significant |
| Gal-9 | 8.29E-01 | 9.95E-01 | Non-significant |
| SCF | 8.69E-01 | 9.95E-01 | Non-significant |
| FGF-21 | 6.89E-01 | 9.95E-01 | Non-significant |
| GH | 8.47E-01 | 9.95E-01 | Non-significant |
| PAPPA | 9.14E-01 | 9.95E-01 | Non-significant |
| DECR1 | 9.22E-01 | 9.95E-01 | Non-significant |
| MERTK | 9.58E-01 | 9.95E-01 | Non-significant |
| KIM1 | 7.98E-01 | 9.95E-01 | Non-significant |
| HO-1 | 7.22E-01 | 9.95E-01 | Non-significant |
| XCL1 | 7.09E-01 | 9.95E-01 | Non-significant |
| PSGL-1 | 8.84E-01 | 9.95E-01 | Non-significant |
| CCL17 | 9.84E-01 | 9.95E-01 | Non-significant |
| CCL3 | 8.47E-01 | 9.95E-01 | Non-significant |
| MMP7 | 9.67E-01 | 9.95E-01 | Non-significant |
| Dkk-1 | 8.05E-01 | 9.95E-01 | Non-significant |
| LPL | 7.99E-01 | 9.95E-01 | Non-significant |
| AGRP | 7.93E-01 | 9.95E-01 | Non-significant |
| GDF-2 | 9.61E-01 | 9.95E-01 | Non-significant |
| THPO | 8.87E-01 | 9.95E-01 | Non-significant |
| MARCO | 6.37E-01 | 9.95E-01 | Non-significant |
| MMP12 | 6.18E-01 | 9.95E-01 | Non-significant |
| PD-L2 | 6.39E-01 | 9.95E-01 | Non-significant |
| CTSL1 | 9.20E-01 | 9.95E-01 | Non-significant |
| TGM2 | 7.56E-01 | 9.95E-01 | Non-significant |
| HSP 27 | 6.54E-01 | 9.95E-01 | Non-significant |
| CD4 | 8.54E-01 | 9.95E-01 | Non-significant |
| NEMO | 9.43E-01 | 9.95E-01 | Non-significant |
| PARP-1 | 6.27E-01 | 9.95E-01 | Non-significant |
| HAOX1 | 9.64E-01 | 9.95E-01 | Non-significant |
| CD84 | 9.96E-01 | 9.96E-01 | Non-significant |

*Adjusted for mass significance using Benjamini-Hochberg method.
Threshold = Adjusted p-value < 0.05

**Supplementary Figure 1: Directed acyclic graph for the selection of covariates in the analyses**

**Reference for the relationship between age and CAD**

Bauersachs R, Zeymer U, Brière JB, Marre C, Bowrin K, Huelsebeck M. Burden of Coronary Artery Disease and Peripheral Artery Disease: A Literature Review. Cardiovasc Ther. 2019 Nov 26;2019:8295054.

**Reference for the relationship between age and BMI**

Chooi YC, Ding C, Magkos F. The epidemiology of obesity. Metabolism. 2019 Mar;92:6-10. doi: 10.1016/j.metabol.2018.09.005. Epub 2018 Sep 22.

**Reference for the relationship between age and diabetes type 2**

Zheng Y, Ley SH, Hu FB. Global aetiology and epidemiology of type 2 diabetes mellitus and its complications. Nat Rev Endocrinol. 2018 Feb;14(2):88-98. doi: 10.1038/nrendo.2017.151. Epub 2017 Dec 8.

**Reference for the relationship between age and hypertension**

Oliveros E, Patel H, Kyung S, Fugar S, Goldberg A, Madan N, Williams KA. Hypertension in older adults: Assessment, management, and challenges. Clin Cardiol. 2020 Feb;43(2):99-107. doi: 10.1002/clc.23303. Epub 2019 Dec 11. PMID: 31825114; PMCID: PMC7021657.

**Reference for the relationship between age and smoking**

World Health Organization. WHO global report on trends in prevalence of tobacco smoking 2000–2025. 3rd ed. Geneva: World Health Organization; 2019.

**Reference for the relationship between age and heart failure**

McDonagh TA, Metra M, Adamo M, Gardner RS, Baumbach A, Böhm M, Burri H, Butler J, Čelutkienė J, Chioncel O, Cleland JGF, Coats AJS, Crespo-Leiro MG, Farmakis D, Gilard M, Heymans S, Hoes AW, Jaarsma T, Jankowska EA, Lainscak M, Lam CSP, Lyon AR, McMurray JJV, Mebazaa A, Mindham R, Muneretto C, Francesco Piepoli M, Price S, Rosano GMC, Ruschitzka F, Kathrine Skibelund A; ESC Scientific Document Group. 2021 ESC Guidelines for the diagnosis and treatment of acute and chronic heart failure. Eur Heart J. 2021 Sep 21;42(36):3599-3726. doi: 10.1093/eurheartj/ehab368. Erratum in: Eur Heart J. 2021 Oct 14;: PMID: 34447992.

Bui AL, Horwich TB, Fonarow GC. Epidemiology and risk profile of heart failure. Nat Rev Cardiol. 2011 Jan;8(1):30-41.

**References for the relationship between age and lung function:**

Talaminos Barroso A, Márquez Martín E, Roa Romero LM, Ortega Ruiz F. Factors Affecting Lung Function: A Review of the Literature. Arch Bronconeumol (Engl Ed). 2018 Jun;54(6):327-332. English, Spanish.

**___________________________________________________________________________________**

**Reference for the relationship between sex and CAD**

Mack M, Gopal A. Epidemiology, Traditional and Novel Risk Factors in Coronary Artery Disease. Heart Fail Clin. 2016 Jan;12(1):1-10.

**Reference for the relationship between sex and BMI**

Chooi YC, Ding C, Magkos F. The epidemiology of obesity. Metabolism. 2019 Mar;92:6-10. doi: 10.1016/j.metabol.2018.09.005. Epub 2018 Sep 22.

**Reference for the relationship between sex and diabetes type 2**

Shepard BD. Sex differences in diabetes and kidney disease: mechanisms and consequences. Am J Physiol Renal Physiol. 2019 Aug 1;317(2):F456-F462. doi: 10.1152/ajprenal.00249.2019. Epub 2019 Jun 26.

**Reference for the relationship between sex and hypertension**

Di Giosia P, Giorgini P, Stamerra CA, Petrarca M, Ferri C, Sahebkar A. Gender Differences in Epidemiology, Pathophysiology, and Treatment of Hypertension. Curr Atheroscler Rep. 2018 Feb 14;20(3):13. doi: 10.1007/s11883-018-0716-z. PMID: 29445908.

**Reference for the relationship between sex and smoking**

L. Greaves. Sifting the evidence: gender and tobacco control. World Health Organization, Department of Gender, Women and Health, Geneva (2007)

World Health Organization. WHO global report on trends in prevalence of tobacco smoking 2000–2025. 3rd ed. Geneva: World Health Organization; 2019.

**Reference for the relationship between sex and heart failure**

McDonagh TA, Metra M, Adamo M, Gardner RS, Baumbach A, Böhm M, Burri H, Butler J, Čelutkienė J, Chioncel O, Cleland JGF, Coats AJS, Crespo-Leiro MG, Farmakis D, Gilard M, Heymans S, Hoes AW, Jaarsma T, Jankowska EA, Lainscak M, Lam CSP, Lyon AR, McMurray JJV, Mebazaa A, Mindham R, Muneretto C, Francesco Piepoli M, Price S, Rosano GMC, Ruschitzka F, Kathrine Skibelund A; ESC Scientific Document Group. 2021 ESC Guidelines for the diagnosis and treatment of acute and chronic heart failure. Eur Heart J. 2021 Sep 21;42(36):3599-3726. doi: 10.1093/eurheartj/ehab368. Erratum in: Eur Heart J. 2021 Oct 14;: PMID: 34447992.

**Reference for the relationship between sex and lung function**

Talaminos Barroso A, Márquez Martín E, Roa Romero LM, Ortega Ruiz F. Factors Affecting Lung Function: A Review of the Literature. Arch Bronconeumol (Engl Ed). 2018 Jun;54(6):327-332. English, Spanish.

**Reference for the relationship between sex and height**

Samaras TT. Should we be concerned over increasing body height and weight? Exp Gerontol. 2009 Jan-Feb;44(1-2):83-92. - 5. How do women’s height and life expectancy compare to men

**___________________________________________________________________________________**

**Reference for the relationship between BMI and CAD**

Flint AJ, Rexrode KM, Hu FB, et al. Body mass index, waist circumference, and risk of coronary heart disease: a prospective study among men and women. Obes Res Clin Pract. 2010;4(3):e171-e181. doi:10.1016/j.orcp.2010.01.001

**Reference for the relationship between BMI and diabetes type 2**

Zheng Y, Ley SH, Hu FB. Global aetiology and epidemiology of type 2 diabetes mellitus and its complications. Nat Rev Endocrinol. 2018 Feb;14(2):88-98. doi: 10.1038/nrendo.2017.151. Epub 2017 Dec 8.

**Reference for the relationship between BMI and heart failure**

McDonagh TA, Metra M, Adamo M, Gardner RS, Baumbach A, Böhm M, Burri H, Butler J, Čelutkienė J, Chioncel O, Cleland JGF, Coats AJS, Crespo-Leiro MG, Farmakis D, Gilard M, Heymans S, Hoes AW, Jaarsma T, Jankowska EA, Lainscak M, Lam CSP, Lyon AR, McMurray JJV, Mebazaa A, Mindham R, Muneretto C, Francesco Piepoli M, Price S, Rosano GMC, Ruschitzka F, Kathrine Skibelund A; ESC Scientific Document Group. 2021 ESC Guidelines for the diagnosis and treatment of acute and chronic heart failure. Eur Heart J. 2021 Sep 21;42(36):3599-3726. doi: 10.1093/eurheartj/ehab368. Erratum in: Eur Heart J. 2021 Oct 14;: PMID: 34447992.

**Reference for the relationship between BMI and hypertension**

Smith KB, Smith MS. Obesity Statistics. Prim Care. 2016 Mar;43(1):121-35, ix. doi: 10.1016/j.pop.2015.10.001. Epub 2016 Jan 12. PMID: 26896205.

**___________________________________________________________________________________**

**Reference for the relationship between smoking and CAD**

Ambrose JA, Barua RS. The pathophysiology of cigarette smoking and cardiovascular disease: an update. J Am Coll Cardiol. 2004 May 19;43(10):1731-7.

**Reference for the relationship between smoking and hypertension**

Williams B, Mancia G, Spiering W, Agabiti Rosei E, Azizi M, Burnier M, Clement DL, Coca A, de Simone G, Dominiczak A, Kahan T, Mahfoud F, Redon J, Ruilope L, Zanchetti A, Kerins M, Kjeldsen SE, Kreutz R, Laurent S, Lip GYH, McManus R, Narkiewicz K, Ruschitzka F, Schmieder RE, Shlyakhto E, Tsioufis C, Aboyans V, Desormais I; ESC Scientific Document Group. 2018 ESC/ESH Guidelines for the management of arterial hypertension. Eur Heart J. 2018 Sep 1;39(33):3021-3104. doi: 10.1093/eurheartj/ehy339. Erratum in: Eur Heart J. 2019 Feb 1;40(5):475. PMID: 30165516.

**Reference for the relationship between smoking and lung function**

Beck GJ, Doyle CA, Schachter EN. Smoking and lung function. Am Rev Respir Dis. 1981 Feb;123(2):149-55.

Tantisuwat A, Thaveeratitham P. Effects of smoking on chest expansion, lung function, and respiratory muscle strength of youths. J Phys Ther Sci. 2014 Feb;26(2):167-70.

**Reference for the relationship between smoking and heart failure**

Bui AL, Horwich TB, Fonarow GC. Epidemiology and risk profile of heart failure. Nat Rev Cardiol. 2011 Jan;8(1):30-41.

**Reference for the relationship between smoking and diabetes type 2**

Zheng Y, Ley SH, Hu FB. Global aetiology and epidemiology of type 2 diabetes mellitus and its complications. Nat Rev Endocrinol. 2018 Feb;14(2):88-98. doi: 10.1038/nrendo.2017.151. Epub 2017 Dec 8.

**___________________________________________________________________________________**

**References for the relationship between hypertension and lung function**

Müllerova H, Agusti A, Erqou S, Mapel DW. Cardiovascular comorbidity in COPD: systematic literature review. Chest. 2013 Oct;144(4):1163-1178. doi: 10.1378/chest.12-2847. PMID: 23722528.

Fisk M, McEniery CM, Gale N, Mäki-Petäjä K, Forman JR, Munnery M, Woodcock-Smith J, Cheriyan J, Mohan D, Fuld J, Tal-Singer R, Polkey MI, Cockcroft JR, Wilkinson IB; ERICA Consortium and ACCT Investigators. Surrogate Markers of Cardiovascular Risk and Chronic Obstructive Pulmonary Disease: A Large Case-Controlled Study. Hypertension. 2018 Mar;71(3):499-506. doi: 10.1161/HYPERTENSIONAHA.117.10151. Epub 2018 Jan 22. Erratum in: Hypertension. 2018 Sep;72(3):e30.

**Reference for the relationship between hypertension and heart failure**

Bui AL, Horwich TB, Fonarow GC. Epidemiology and risk profile of heart failure. Nat Rev Cardiol. 2011 Jan;8(1):30-41.

**Reference for the relationship between hypertension and CAD**

Malakar AK, Choudhury D, Halder B, Paul P, Uddin A, Chakraborty S. A review on coronary artery disease, its risk factors, and therapeutics. J Cell Physiol. 2019 Aug;234(10):16812-16823. doi: 10.1002/jcp.28350. Epub 2019 Feb 20. PMID: 30790284.

**___________________________________________________________________________________**

**References for the relationship between diabetes and lung function:**

Walter RE, Beiser A, Givelber RJ, O’Connor GT, Gottlieb DJ. Association between glycemic state and lung function: the Framingham Heart Study. Am J Respir Crit Care Med. 2003;167(6):911–6.

Yeh HC, Punjabi NM, Wang NY, Pankow JS, Duncan BB, Cox CE, Selvin E, Brancati FL. Cross-sectional and prospective study of lung function in adults with type 2 diabetes: the Atherosclerosis Risk in Communities (ARIC) study. Diabetes Care. 2008;31(4):741–6.

Lange P, Groth S, Kastrup J, Mortensen J, Appleyard M, Nyboe J, Jensen G, Schnohr P. Diabetes mellitus, plasma glucose and lung function in a cross-sectional population study. Eur Respir J. 1989;2(1):14–9.

Litonjua AA, Lazarus R, Sparrow D, Demolles D, Weiss ST. Lung function in type 2 diabetes: the Normative Aging Study. Respir Med. 2005;99(12):1583–90.

Chance WW, Rhee C, Yilmaz C, Dane DM, Pruneda ML, Raskin P, Hsia CC. Diminished alveolar microvascular reserves in type 2 diabetes reflect systemic microangiopathy. Diabetes Care. 2008;31(8):1596–601.

**Reference for the relationship between diabetes and heart failure**

Lehrke M, Marx N. Diabetes Mellitus and Heart Failure. Am J Cardiol. 2017 Jul 1;120(1S):S37-S47. doi: 10.1016/j.amjcard.2017.05.014. Epub 2017 May 30.

Bui AL, Horwich TB, Fonarow GC. Epidemiology and risk profile of heart failure. Nat Rev Cardiol. 2011 Jan;8(1):30-41.

**Reference for the relationship between diabetes and CAD**

Malakar AK, Choudhury D, Halder B, Paul P, Uddin A, Chakraborty S. A review on coronary artery disease, its risk factors, and therapeutics. J Cell Physiol. 2019 Aug;234(10):16812-16823. doi: 10.1002/jcp.28350. Epub 2019 Feb 20. PMID: 30790284.

**Reference for the relationship between height and lung function**

Bhatti U, Rani K, Memon MQ. Variation in lung volumes and capacities among young males in relation to height. J Ayub Med Coll Abbottabad. 2014 Apr-Jun;26(2):200-2.

**Reference for the relationship between height and heart failure**

Rosenbush SW, Parker JM. Height and heart disease. Rev Cardiovasc Med. 2014;15(2):102-8. PMID: 25051127.

**Reference for the relationship between height and CAD**

Rosenbush SW, Parker JM. Height and heart disease. Rev Cardiovasc Med. 2014;15(2):102-8. PMID: 25051127.

Nelson CP, Hamby SE, Saleheen D, Hopewell JC, Zeng L, Assimes TL, Kanoni S, Willenborg C, Burgess S, Amouyel P, Anand S, Blankenberg S, Boehm BO, Clarke RJ, Collins R, Dedoussis G, Farrall M, Franks PW, Groop L, Hall AS, Hamsten A, Hengstenberg C, Hovingh GK, Ingelsson E, Kathiresan S, Kee F, König IR, Kooner J, Lehtimäki T, März W, McPherson R, Metspalu A, Nieminen MS, O'Donnell CJ, Palmer CN, Peters A, Perola M, Reilly MP, Ripatti S, Roberts R, Salomaa V, Shah SH, Schreiber S, Siegbahn A, Thorsteinsdottir U, Veronesi G, Wareham N, Willer CJ, Zalloua PA, Erdmann J, Deloukas P, Watkins H, Schunkert H, Danesh J, Thompson JR, Samani NJ; CARDIoGRAM+C4D Consortium. Genetically determined height and coronary artery disease. N Engl J Med. 2015 Apr 23;372(17):1608-18. doi: 10.1056/NEJMoa1404881. Epub 2015 Apr 8. PMID: 25853659; PMCID: PMC4648271.

**References for the relationship between heart failure and lung function:**

Kawakami R, Nakada Y, Hashimoto Y, Ueda T, Nakagawa H, Nishida T, Onoue K, Soeda T, Watanabe M, Saito Y. Prevalence and Prognostic Significance of Pulmonary Function Test Abnormalities in Hospitalized Patients With Acute Decompensated Heart Failure With Preserved and Reduced Ejection Fraction. Circ J. 2021 Apr 16. doi: 10.1253/circj.CJ-20-1069. Epub ahead of print. PMID: 33867406.

Arnett DK, Goodman RA, Halperin JL, et al. . AHA/ACC/HHS strategies to enhance application of clinical practice guidelines in patients with cardiovascular disease and comorbid conditions: from the American Heart Association, American College of Cardiology, and U.S. Department of Health and Human Services. *J Am Coll Cardiol* 2014;64:1851–6. 10.1016/j.jacc.2014.07.012

**Reference for the relationship between heart failure and CAD**

Bui AL, Horwich TB, Fonarow GC. Epidemiology and risk profile of heart failure. Nat Rev Cardiol. 2011 Jan;8(1):30-41.
